# A validation study of public health knowledge, skills, social responsibility and applied learning

**DOI:** 10.5116/ijme.5b1b.910d

**Published:** 2018-06-22

**Authors:** Dana Vackova, Coco K. Chen, Juliana N.M. Lui, Janice M. Johnston

**Affiliations:** 1 School of Public Health, LKS Faculty of Medicine, The University of Hong Kong, Hong Kong

**Keywords:** Public health, questionnaire development, validity and reliability, undergraduate medical education, ASPPH undergraduate leaning outcomes model

## Abstract

**Objectives:**

To design and validate a questionnaire to measure medical students’ Public Health (PH) knowledge, skills, social responsibility and applied learning as indicated in the four domains recommended by the Association of Schools & Programmes of Public Health (ASPPH).

**Methods:**

A cross-sectional study was conducted to develop an evaluation tool for PH undergraduate education through item generation, reduction, refinement and validation. The 74 preliminary items derived from the existing literature were reduced to 55 items based on expert panel review which included those with expertise in PH, psychometrics and medical education, as well as medical students. Psychometric properties of the preliminary questionnaire were assessed as follows: frequency of endorsement for item variance; principal component analysis (PCA) with varimax rotation for item reduction and factor estimation; Cronbach’s Alpha, item-total correlation and test-retest validity for internal consistency and reliability.

**Results:**

PCA yielded five factors: PH Learning Experience (6 items); PH Risk Assessment and Communication (5 items); Future Use of Evidence in Practice (6 items); Recognition of PH as a Scientific Discipline (4 items); and PH Skills Development (3 items), explaining 72.05% variance. Internal consistency and reliability tests were satisfactory (Cronbach’s Alpha ranged from 0.87 to 0.90; item-total correlation > 0.59). Lower paired test-retest correlations reflected instability in a social science environment.

**Conclusions:**

An evaluation tool for community-centred PH education has been developed and validated. The tool measures PH knowledge, skills, social responsibilities and applied learning as recommended by the internationally recognised Association of Schools & Programmes of Public Health (ASPPH).

## Introduction

Multidisciplinary Public Health (PH) represents an academic and clinical discipline that focuses on disease prevention and human health promotion through organised efforts and informed choices by individuals, communities and society as a whole.  While there is broad consensus about the importance of PH education, medical curricula vary amongst countries and faculties in the depth and breadth of PH teaching and learning through differences in pedagogical approaches, core competencies, and levels of integration of undergraduate PH in core medical curricula.^1^^-^^6^ Some medical educators and health care professionals comment that undergraduate medical students are not readily equipped with skills to analyse or appraise PH issues as they focus primarily on case diagnosis and treatment, while others support global policy initiatives to equip fresh medical graduates with both clinical and PH competences.^7^^,^^3^^,^^8^^,^^9^

Despite the above debates, it is in unified agreement that the content of undergraduate PH programmes should address the local community needs, lifestyle and environment, risk communication and disease trends, and policies and recommendations.^10^^-^^13^ To facilitate integration of community-based PH activities into different undergraduate curricula or co-curricular activities, the Association of Schools & Programmes of Public Health (ASPPH)  has developed an undergraduate learning outcomes model comprising four main domains, namely,  1) knowledge of human cultures and the physical and natural world as it relates to individual an population health, 2) intellectual and practical skills, 3) personal and social responsibility, and  4) integrative and applied learning.^13^     These domains are consistent with the increased need for community-centred pedagogy for medical education to encourage students in addressing community needs.^13^^-^^18^

The obvious challenges in undergraduate PH teaching also persist in the assessment strategies and methods applied in PH programme evaluation, where the universal questionnaires provided by universities do not specify in assessing students’ perception on PH teaching and multidisciplinary PH competences development.^6^^,^^19^^-^^21^  In addition, previous studies showed that there is a sparse number of instruments in measuring the effectiveness of PH teaching and learning, in which existing ones non-comprehensively focus on a small subset of PH teaching and learning factors and fail to address internationally recognised PH domains.^8^^,^^9^^,^^20^^,^^22^^,^^23^ Thus, a standardized multi-dimensional questionnaire measuring the self-reported development of PH core competencies of local and global importance is essential amongst medical educators.  Therefore, this study aims to design and validate a questionnaire to measure medical students’ perceptions of undergraduate PH teaching and learning in all four main domains recommended by the Association of Schools & Programmes of Public Health (ASPPH).

## Methods

### Study design and participants

The cross-sectional study on development and validation of a new instrument assessing student progress on gaining public health knowledge, skills, social responsibility and applied learning comprised of two stages: (1) item generation and item reduction and (2) assessment of psychometric properties. The design and development process was conducted according to the following two Association for Medical Education in Europe (AMEE) Guides: (1) Introduction to research in medical education and (2) Quantitative and qualitative methods in medical education that included qualitative and quantitative research designs and psychometric studies.^24^^-^^26^ The ASPPH Undergraduate Learning Outcomes Model was incorporated into the study to provide the framework for item generation and content validity using four ASPPH domains: (1) knowledge of human cultures and the physical and natural world as it relates to individual and population health, (2) intellectual and practical skills, (3) personal and social responsibility, and (4) integrative and applied learning. ^13^ Content validation on item relevance was carried out by experts in PH, psychometrics and curriculum development.

Years three and four undergraduate medical students who have already completed their PH curriculum in pre-clinical and clinical settings were invited to participate in the study by e-mail and in-person during class sessions and tutorials. Year 4 medical students (N=20 of 198; female 50%, male 50%) were randomly selected to join the validation expert team. Year 3 medical students (N=133 of 204; female 71%, male 29.0%) completed the preliminary questionnaire. Consequent Year 3 students completed test-retest (N=90 of 201, female 46%, male 54%).

The study was carried out according to the Belmont report ethical considerations. All participants were assured that their participation is voluntary and confidential, no harm would be afflicted upon them during their study, and their refusal in participating in the study will have no impact on their course assessments or grades.^25^ Written consent was obtained from all participants. Each participant was assigned a unique study number, and only the research assistant had access to the master identification file. Ethical approval was obtained from the Institutional Review Board of the University of Hong Kong/Hospital Authority Hong Kong West Cluster.

### Setting

The study was conducted at the Li Ka Shing Faculty of Medicine, the University of Hong Kong (HKU), where multidisciplinary PH courses are well- integrated into the Bachelor of Medicine Bachelor of Surgery (MBBS) programme.^27^

### Data collection methods

**Stage 1: Item selection process and content validity**

A comprehensive literature search via Pubmed database and grey literature was completed by two researchers to identify individual items, and validated instruments and methods to evaluate PH teaching and learning in medical curricula (undergraduate or otherwise).  The literature search identified a broad list of potential items (N=74) to be considered for inclusion in the questionnaire. Experts (N=8) held meetings in mapping the generated items to the most relevant ASPPH domain, under an assumption that knowledge and skills covered in the first two domains were considered as prerequisites for the higher level of learning process reflected in domains 3 and 4.

### Procedures

The following three-step interactive approach was adopted for item reduction: ^23^

Firstly, PH experts (N=8) who were knowledgeable with the undergraduate curriculum were invited to rate all items by their relevance and appropriateness to public health undergraduate education and PH learning outcomes on a five-point Likert scale (1=most relevant to 5= not relevant). Items with an average score of 3 or above were recommended for inclusion. Secondly, MBBS Year 4 students (N=20) were interviewed using semi-structured questions to assess the comprehensibility, relevance and usefulness of the questionnaire items and to reduce items further. The interviews also elicited students’ feedback on their undergraduate PH teaching and the anticipated impact of PH teaching and learning on their future clinical practice. Finally, experts in psychometrics, questionnaire development, and PH (N=6) provided feedback on the questionnaire content validity, response scale selection, comprehensiveness and comprehensibility.

The resulting preliminary questionnaire contained 55 items, where 2 were open-ended items and 53 items used a six-point Likert scale (1=strongly disagree to 6=strongly agree) to measurably intensify the degree of students’ cognitive involvement and their commitment to either the positive or negative end of the rating scale.^28^

**Stage 2: Psychometric assessment and item refinement**

The preliminary 55-item questionnaire was distributed to Year 3 students (N=133 of 204, response rate = 65%) during a whole class session to conduct psychometric assessment and further refine the items. 

### Data analysis

Data analysis was carried out in SPSS for Windows, Version 20. Descriptive statistics including mean and variance were used to summarize the data initially. Subsequently, psychometric properties of the questionnaire were assessed, where the frequency of endorsement evaluated item variance, and the Principal Component Analysis (PCA) with varimax rotation was used for item reduction and factor estimation. Furthermore, Cronbach’s Alpha, item-total correlation, and test-retest reliability were performed to evaluate the internal consistency of the questionnaire.

The frequency of endorsement was assessed by splitting the response into two categories: ‘disagree’ (original rating =1, 2, and 3) and ‘agree’ (original rating = 4, 5, and 6), and then calculating the proportion of dichotomous responses. Items with very high (95%) or very low (5%) endorsement frequency were discarded.^29^^,^^30^ To examine the underlying structure of the questionnaire, the PCA with varimax rotation was used. Sampling adequacy was confirmed if Kaiser-Mayer-Olkin (KMO) test yielded a value greater than 0.60 and significant Bartlett’s test of sphericity had a p-value equal to or smaller than 0.05 (p ≤ 0.05).^31^ Items were retained if factor loading was greater than 0.40 without cross-loading.^32^ The guiding criteria used to determine the number of factors was the Eigenvalue greater than 1 (Kaiser’s criterion), the Scree Plot, the occurrence of more than three items per factor and total variance that would explain approximately 75% of the variance using the least number of factors.^33^ Additionally, Cronbach’s Alpha (Alpha >0.70), item-total correlation, and test-retest reliability were used to confirm internal consistency.^32^ Items in the same factor that scored <0.2 in the inter-item correlation were discarded.^29^

The 24-item finalized questionnaire was distributed to the consequent Year 3 students who completed the questionnaire twice for test re-test reliability (N=90 of 201, response rate = 44.77%).  Paired sample correlation coefficients were calculated to assess test-retest reliability.

## Results

### Construct validity

In the item variance test, 52 of 53 items satisfied the pre-set frequency of endorsement level of 20% - 80%. The remaining item was reworded but retained due to its conceptual importance. The KMO index was 0.85 and Bartlett’s test of sphericity was significant (χ^2^ (378) = 2676.35, p<0.001).  In total, 29 of 53 (55%) items did not meet the factor loading criteria. To confirm the inclusion or exclusion of each of these items, factor analysis was repeated with each item being removed one by one.^32^

The principal component analysis yielded a 24-item 5-factor structure, confirmed by the scree plot, explaining 72.05% of the variance (Table 1).  Factor 1: PH learning experience with 6 items explained 36.12% of the total variance; Factor 2: PH risk assessment and communication with 5 items explained 13.19% of the variance; Factor 3: Future use of evidence in practice with 6 items explained 9.43% of the variance; Factor 4: Recognition of PH as a scientific discipline with 4 items explained 7.19% of the variance; and Factor 5: PH skills development with 3 items explained 6.12% of the variance.

**Table 1:** Principle component analysis with communalities of each item (N=24)

Item	F1	F2	F3	F4	F5	Extraction	Mean	SD
1	0.82					0.77	4.07	0.84
2	0.80					0.70	4.00	0.88
3	0.76					0.65	3.82	1.04
4	0.75					0.72	4.06	0.88
5	0.67					0.61	4.24	0.88
6	0.61					0.59	3.79	0.98
7		0.86				0.82	4.34	0.81
8		0.80				0.75	4.29	0.76
9		0.76				0.71	4.35	0.85
10		0.74				0.72	4.38	0.75
11		0.73				0.70	4.36	0.72
12			0.84			0.79	4.57	0.78
13			0.83			0.73	4.61	0.69
14			0.77			0.67	4.51	0.73
15			0.76			0.65	4.34	0.69
16			0.60			0.64	4.34	0.71
17			0.59			0.60	3.52	0.97
18				0.91		0.86	3.31	0.99
19				0.86		0.75	3.36	1.06
20				0.82		0.83	3.57	1.07
21				0.77		0.69	4.16	0.71
22					0.80	0.76	4.07	0.76
23					0.79	0.82	4.09	0.82
24					0.76	0.78	4.07	0.84
% Variance	36.12	13.19	9.43	7.19	6.12			

Note: Factor loading less than 0.40 were removed.

### Measurement reliability

Cronbach’s Alpha ranged from 0.87 to 0.90. The PH learning experience (Factor 1) consisted of 6 items (Alpha = 0.90); The PH risk assessment and communication (Factor 2) consisted of 5 items (Alpha = 0. 90); the Future use of evidence (Factor 3) consisted of 6 items (Alpha = 0.88), the Recognition of PH as a Scientific discipline (Factor 4) consisted of 4 items (Alpha = 0.87); and PH skill development (Factor 5) consisted of 3 items (Alpha=0.87). All factors achieved acceptable item-total correlation ranging from 0.59 to 0.85. The final validated 24-item questionnaire was distributed for test re-test reliability with paired sample correlation coefficients ranging from 0.26 to 0.61 (Table 2).

**Table 2:** Reliability and stability analyses performed on the final version of the questionnaire

Factors		Number of items	Range of item-total correlation	Cronbach's alpha coefficient	Paired test-retest correlation
1	PH teaching and learning	6	0.68 - 0.82	0.90	0.37^**^
2	PH Risk assessment a communication	5	0.72 - 0.83	0.90	0.61^**^
3	Future use of evidence in practice	6	0.65 - 0.79	0.88	0.54^**^
4	Perception of PH as a scientific discipline	4	0.59 - 0.85	0.87	0.50^**^
5	PH Skills Development	3	0.73 - 0.78	0.87	0.26^*^

*p<0.05;**p<0.001

**Table 3:** Mapping of questionnaire items (N=24) to the ASPPH four main domains

Factors	Items	ASPPH Domain 1	ASPPH Domain 2	ASPPH Domain 3	ASPPH Domain 4
PH learning experience	1. Overall, Public Health (PH) teaching was effective in helping me achieve the PH core competencies	NA	NA	NA	NA
	2. The assessment criteria were appropriate in relation to PH learning outcomes	NA	NA	NA	NA
	3. PH lectures inspired me to learn more about PH disciplines	NA	NA	NA	NA
	4. I was able to cope with the PH workload	NA	NA	NA	NA
	5. The PH tutors were competent in PH teaching	NA	NA	NA	NA
	6. The PH teaching was well organized	NA	NA	NA	NA
PH risk assessment and communication	7. I will adopt reliable and consistent inter- and intra-professional communication skills with health policy makers			v	
	8. I will adopt reliable and consistent inter- and intra-professional communication skills with media			v	
	9. I will communicate health information to policymakers			v	
	10. I am able to assess the role of lifestyle factors on individual/population health			v	v
	11. I am able to assess the role of environment on population and health			v	v
Future use of evidence in practice	12. I will interpret clinical research for patient care and for population health			v	
	13. I will communicate health information to individual patients			v	
	14. I will adopt reliable and consistent inter- and intra-professional communication skills with other healthcare professionals		v		
	15. I will communicate health information to healthcare professionals				v
	16. I will apply my critical appraisal skills to the evaluation of research papers		v		
	17. I will use my literature search skills to support my application of evidence-based medicine				v
Recognition of PH as a scientific discipline	18. The application of behavioral science to medicine is common sense, not a scientific discipline	v			
	19. The application of PH principles to clinical practice is common sense, not a scientific discipline	v			
	20. The application of health promotion principles to medicine is common sense, not a scientific discipline	v			
	21. Medicine is about people, not PH evidence-based practice and statistics	v			
PH skills development	22. Compared to one year ago, PH teaching has enhanced my skills to apply economic principles in clinical decision making			v	v
	23. Compared to one year ago, PH teaching has enhanced my skills to promote the health of individual and public in modern clinical practice			v	v
	24. Compared to one year ago, PH teaching has enhanced my skills to address environmental PH problems			v	v

Notes: NA: item is not applicable for mapping; v: item was mapped to the strongest ASPPH domain assuming that knowledge and skills covered in the first two domains were prerequisites for a higher level of learning process reflected in domains 3 or 4. Domain 1: Knowledge of human cultures and the physical and natural world as it relates to the individual and population health; Domain 2: Intellectual and practical skills; Domain 3: Personal and social responsibility; Domain 4: Integrative and applied learning^13^

Mapping of the validated questionnaire items (N=24) to the four ASPPH domains

All items except 6 loaded on PH learning experience (Factor 1) were mapped to the most relevant domains, under the assumption that the first two domains covering knowledge and skills were prerequisites for higher level of learning process reflected in domains 3 and 4. Factors 2 and 5 covered mainly higher domains 3 and 4, whereas items loaded on Factor 3 and 4 were mainly aligned to the knowledge and skills (Table 3).

## Discussion

As presented in this paper, a questionnaire measuring medical students’ perceptions of their PH knowledge, skills, social responsibility and applied learning was developed with satisfactory validity and reliability. The underlying 4-domain ASPPH Undergraduate Learning Outcomes Model was adopted in this study as a theoretical framework for the reason that it enables undergraduate students to become more active participants in their community health through access to PH education integrated into different curricular and co-curricular activities. Although the ASPPH framework is not comprehensive or prescriptive, it represents the key trends in pedagogy that emphasise innovative teaching methods and problem-solving through interactive, experiential and applied learning. Additionally, the ASPPH framework includes recommendations for an educated citizenry and student’s contribution to the quality of life globally and locally.^13^

Year 3 and 4 undergraduate medical students participated in the validation process, because they had previous experience with PH teaching and learning in both pre-clinical and clinical settings, therefore demonstrating better ability to comprehend knowledge, skills, social responsibilities and applied learning in PH.

Possible biases in rating the relevance and item reduction were addressed by inviting experts from different PH sub-disciplines and by the three-step interactive approach. Subject to their area of PH expertise, all experts provided their opinion on the items’ appropriateness for the undergraduate level of PH education and competences. Although there was a consensus on the selected items for the preliminary questionnaire, the experts’ views on the best format for the response scale varied from 5 to 7-point Likert scale to more complex formats, for example choosing answers among different response options. Despite some research studies showing that researchers may have a preference for 5-point scale, our expert panel in psychometrics preferred an even number of ratings in the scale to eliminate the midpoint as a proxy for "no opinion" and intensify the students’ commitment to the positive or negative end of scale.^28^

The questionnaire was found to have satisfactory internal validity and reliability.^29^^,^^30^ After exploratory factor analysis, the final version of the questionnaire contained 24 items loaded on five factors. The percentage of total variance of 72.05% was acceptable considering that there is usually no absolute threshold adopted for all applications in social science and even 60% total variance may be considered as satisfactory.^32^ The questionnaire has excellent internal consistency, as measured by Cronbach’s alpha and item-total correlation. The less than the satisfactory stability of the questionnaire, as measured by paired test-retest correlation coefficient, may be contributed to potential changes in students’ attitudes and perceptions due to exposure to PH teaching during the 2 weeks interval between test-retest. Also, only 90 out of 133 students participated in the re-test, resulting in unstable estimates.

The derived five factors in the questionnaire appeared to be independent, multidimensional, and consistent. All items, except 6 loaded on PH learning experience (Factor 1), were mapped to the four main ASPPH domains framework, i.e., PH knowledge, Intellectual and practical skills, Personal and social responsibilities, and Integrative and applied learning.

PH risk assessment and communication (Factor 2), Future use of evidence in Practice (Factor 3) and PH skills development (Factor 5) cover all four domains through items representing knowledge, inquiry and analysis, critical thinking, local and global engagement and communication, synthesis and advanced accomplishment. Factor 4 measures students’ perceptions of PH as a scientific discipline and is especially suitable for monitoring the changes in attitudes towards PH in longitudinal educational studies exploring scientific foundations and skills necessary for community engagement.

### Limitations of the study

First, the experts and medical students were mainly considering the local needs of undergraduate PH teaching and learning, which may have an impact on the content validity. Second, medical students selected by convenience sampling may not represent the views and perceptions of the entire MBBS community. Age, year of study, gender, previous engagement in community work, relevance to clinical practice and pedagogy style are among some of the factors that may affect medical students’ perception towards Public Health.

Furthermore, the preliminary 55-item questionnaire was not distributed to all MBBS students for validation, and therefore the utility of the questionnaire for evaluating the whole spectrum of the curriculum may be limited.  However, by the end of Year 3, medical students have been exposed to approximately 80% of the undergraduate PH teaching and learning content. As perception and attitudes toward teaching and learning change over time with increasing experience and exposure, it would also be useful to include residents and interns in any future questionnaire validation.

In addition, Cronbach’s alpha was used to measure internal reliability. However, the tau-equivalent assumptions that the items measure a single underlying construct may not be reached especially in social sciences. Thus, Cronbach’s alpha may provide a below average measure for the reliability.^34^ Finally, the reliability and validity of the questionnaire could be tested in other countries or regions to examine the generalization of the internal structure and reliability of the questionnaire.

## Conclusions

The validated questionnaire measures medical students’ perception about undergraduate multidisciplinary Public Health teaching and learning. It measures perceived competencies in all four main domains recommended by the internationally recognised Association of Schools & Programmes of Public Health, including knowledge, practical PH skills, personal and social responsibilities and integrative and applied learning. The questionnaire can be used as one of the evaluation tools in the undergraduate PH curriculum to evaluate changes in students’ perception towards PH and measure PH competences in longitudinal studies.

### Acknowledgements

Authors would like to thank Mr Alex WY Kwan for his enormous administrative support during the questionnaire development and data collection. Our thanks also belong to all PH experts whose valuable opinion was crucial for the questionnaire development. The HKU Teaching Development Grant financially supported this study.

### Conflicts of Interest

The authors declare that they have no conflict of interest.
